# Fatigue Life of 7475-T7351 Aluminum After Local Severe Plastic Deformation Caused by Machining

**DOI:** 10.3390/ma12213605

**Published:** 2019-11-03

**Authors:** Petra Ohnistova, Miroslav Piska, Martin Petrenec, Jiri Dluhos, Jana Hornikova, Pavel Sandera

**Affiliations:** 1 Department of Manufacturing Technology, Faculty of Mechanical Engineering, Brno University of Technology, 616 69 Brno, Czech Republic; piska@fme.vutbr.cz; mpetrenec@gmail.com; 2 TESCAN ORSAY HOLDING a.s., 623 00 Brno, Czech Republic; jiri.dluhos@tescan.com; 3 Central European Institute of Technology, Brno University of Technology, Brno 612 00, Czech Republic; hornikova@fme.vutbr.cz (J.H.); sandera@fme.vutbr.cz (P.S.)

**Keywords:** crack nucleation, fatigue, plastic deformation, surface topography

## Abstract

The fatigue properties of thermo-mechanically treated and machined aluminum alloy 7475-T7351 have been studied. The applied advanced machining strategy induced intensive plastic deformation on the machined surface under defined cutting conditions. Therefore, a detailed study of 3D surface topography was performed. Advanced characterization of the material structure and electron back scattered diffraction mapping of selected chemical phases were performed, as well as energy dispersive X-ray analysis of the surface. Advanced mechanical properties of the material were investigated in situ with a scanning electron microscope that was equipped with a unique tensile fixture. The fatigue results confirmed an evident dispersion of the data, but the mechanism of crack nucleation was established. Fracture surface analysis showed that the cracks nucleated at the brittle secondary particles dispersed in the material matrix. The surface topography of samples that had been machined in wide range of cutting/deformation conditions by milling has not proved to be a decisive factor in terms of the fatigue behavior. The incoherent interface and decohesion between the alumina matrix and the brittle secondary phases proved to significantly affect the ultimate strength of the material. Tool engagement also affected the fatigue resistance of the material.

## 1. Introduction

Aluminum and its alloys are used in a wide range of industrial applications. Duralumins, are employed in the aerospace industry. Due to an ideal combination of low density, high strength, good corrosion resistance, and high resistance to fatigue crack propagation, these special alloys take precedence over other structural materials [1]. Aluminum alloys of the 2000 and 7000 series are widely used for primary and secondary aircraft structures, such as frames, spares, and ribs, where any damage has a crucial impact on safety.

The high strength Al-Zn-Mg-Cu 7475 is an alloy with controlled toughness made in the form of sheets and plates, that has an ideal combination of high strength, good fracture toughness, and resistance to fatigue crack propagation. The 7475 alloy has almost 40% greater fracture toughness than the previous version, 7075 [2]. This progress in mechanical properties is a result of the reduction of the content of iron, silicon, and magnesium, and application of thermo-mechanical and heat treatments which achieve a refined grain size [3]. The 7475 alloy, in the form of plates, is usually available in different tempered conditions such as T651, T7351, and T7651 [4]. However, among modifying the chemical composition of the alloys, the mechanical properties of Al-based alloys can also be enhanced via severe plastic deformation (SPD) methods, including high pressure torsion (HPT) [5], equal channel angular pressing (ECAP) and its modifications [6,7,8], or rotary swaging (RS) [9].

As the significant portion of components made of alloy 7475-T7351 are processed by different machining strategies, it is difficult to determine the mechanisms of the severe plastic deformation imposed by the each machining process.

The machining process is generally characterized by chip formation as the direct result of the force interactions between the tool and the workpiece. To achieve chip separation, the stress applied between the tool and the workpiece in the chip formation zone must exceed the ultimate strength of the workpiece material. This force interaction is highly dependent on the studied material, the geometry and material of the tool, the cutting environment, and on the defined cutting conditions. However, during chip formation, three zones of plastic deformation can be always observed: (1) a primary deformation zone in front of the cutting tool edge with extreme shear deformation (*γ* = 2–5) and deformation rates (10^3^–10^8^ s^−1^), (2) a secondary deformation zone between the chip and the tool rake face, and (3) a tertiary deformation zone between the machined surface and the tool flank face. In the zone of primary plastic deformation, the chip reaches a high temperature over its entire cross-section (in some cases near to the melting point). As a result of this elevated temperature, the metallurgical and mechanical properties change, the chip softens, the frictional force and the cutting resistance between tool and workpiece decreases, the shear plane angle increases, the chip cross section becomes thinner, and the chip speed increases. In the zone of tertiary plastic deformation, the cutting edge of the tool initiates a stress concentration at the contact zone between the tool and the workpiece [10,11,12]. The machined surface layer is subjected to elastic and plastic deformation at a temperature lower than the temperature of recrystallization. Therefore, material is not melted and any material texture change is permanent and the surface layer is hardened. The imposed force interaction and the thermal load result in residual stress concentration at different depths of the surface layer. These residual stresses can be compressive, increasing the fatigue limit, or tensile, reducing the fatigue limit [13]; they can also alter other mechanical and utility properties [14,15].

As it is well known that nuclei of fatigue cracks are mostly observed on the free surface of the loaded component, all previously described factors have to be taken into consideration while choosing an appropriate machining strategy and machining conditions. Initiation of fatigue cracks on the machined surface is usually closely associated with the combination of severe plastic deformation and the presence of material inclusions or severe plastic deformation and significantly deteriorated surface topography [16].

Ojolo at al. [17] presented results of the four-point bending fatigue testing of a end-milled specimen of alloy 2024. The important fatigue life increase (from 2.67 × 10^3^ to 3.6 × 10^3^ cycles to failure) was observed for samples machined with use of higher cutting speeds (from 3.77 m/min to 48.25 m/min). A decrease of the surface roughness was observed with an increase of the cutting speed, which may be the result of the thermal softening effect due to accumulated heat which caused a temperature rise in the machining zone. The feed speed was described as the most influential factor affecting the fatigue life. The increase of fatigue life was observed with a decrease of the feed speed (from 60 mm/min to 7 mm/min). Surface topography, on the other hand, deteriorated with increase of the feed speed. Ojolo therefore supposed that while using higher feed speeds, the teeth of the end-mill cutter do not perform perfect swiping of the entire surface of the machined zone to make a perfectly smooth surface. It was also observed that an increase in rake angle from 30° to 45° resulted in a better surface finish and increased the fatigue life of the specimens (from 2.53 × 10^3^ to 3.49 × 10^3^ cycles to failure).

Many other studies have been devoted to determining the effect of machining strategies on the fatigue life. Some have demonstrated the influence of the surface topography. The effect of machining and surface integrity on the fatigue life has been summarized by Novovic et al. [18]. They reported that a large dispersion of results has been found in the literature; however, fatigue life increase was mostly observed with decreasing surface roughness. Koster [19] found that for a roughness parameter (*Ra*) between 2.5 and 5 μm, the residual stress imposed by the machining process is the most important factor affecting the fatigue life of structural alloys. Koster [19] also reported that this effect was suppressed by elevated temperature, which allowed relaxation of imposed residual stresses. However, a study of progressive milling technology on the surface topography and fatigue life of aluminum alloy 7475 of Piska et al. [20] showed that in the case of the presence of material inclusions or secondary phases larger than standard topography parameters (such as average roughness of the profile, maximum depth of the valley of the roughness profile, and others), the effect of the surface topography is usually suppressed.

Regarding ever-increasing safety requirements, it is necessary to carefully analyze the effect of the surface quality, including material structure and surface topography together with residual stresses and severe plastic deformation, imposed by the machining process before releasing components into operation.

This study is, therefore, focused on the influence of the different cutting conditions and tool inclination of the face milling strategy applied on the bottom wing panel made of alloy 7475-T7351 with regard to its fatigue life during operational use. The main goal of this study is therefore to define the milling condition range that allows maintaining the optimum balance between the productivity of the production process, the quality of machined surface, and the required fatigue properties.

## 2. Experimental Materials and Methods

### 2.1. Material

The aluminum alloy 7475-T7351 in the form of 70 mm thick plates was used in this study. The heat treatment with designation T7351 denotes solution heat treatment at 470 °C, water quenching, controlled stretching, and artificial ageing (over-aged in two stages: first at 121 °C for 25 h, second at 163 °C for a period of 24–30 h). The average chemical composition of the alloy is presented in Table 1 and its basic mechanical properties are shown in Table 2.

**Figure 1:**
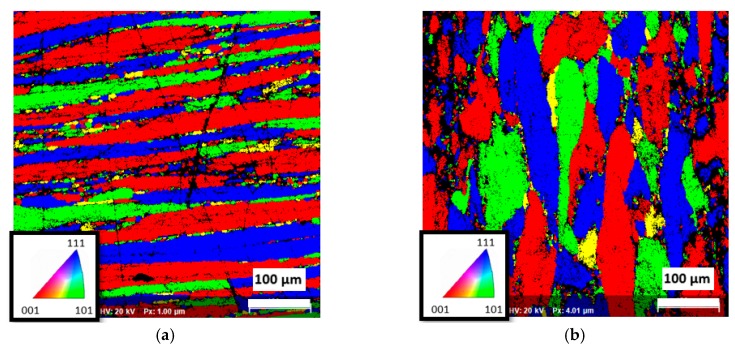
Structure of aluminum alloy 7475-T7351 determined by electron back scattered diffraction mapping (EBSD): (**a**) Longitudinal direction; (**b**) Transversal direction.

The electron back scattered diffraction mapping (EBSD) study showed a heavily deformed structure with high anisotropy and texture of the grains (and very fine subgrains), as shown in Figure 1.

**Figure 2:**
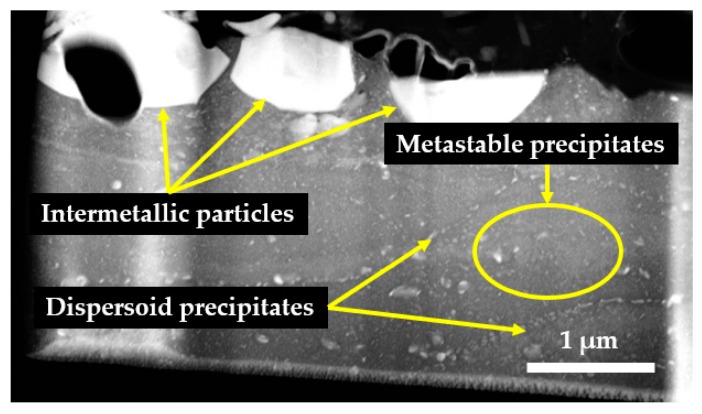
STEM lamella of aluminum alloy 7475-T7351—occurrence of secondary phases.

During STEM (Scanning Transmission Electron Microscopy) lamella analysis, three different secondary phases have been observed in the material matrix, as indicated in Figure 2. Coarse intermetallic particles Al-Cu-Fe (possibly Al_7_Cu_2_Fe) [21,22] and Al-Cr-Fe-Cu-Si in the range from 2 µm up to 20 µm were formed during solidification phase. Precipitated Al-Fe-Si and Al-Mg-Cr dispersoids (possibly Al_12_Fe_3_Si; Al_12_Mg_2_Cr) were formed by solid state precipitation in the grain boundaries. Third, observed secondary phases can be described as fine metastable precipitates in the material matrix (sizes from 2 nm up to 0.6 µm) and these are responsible for strengthening of the alloy (via *GP*, *ή* or *η*) [22,23].

**Figure 3:**
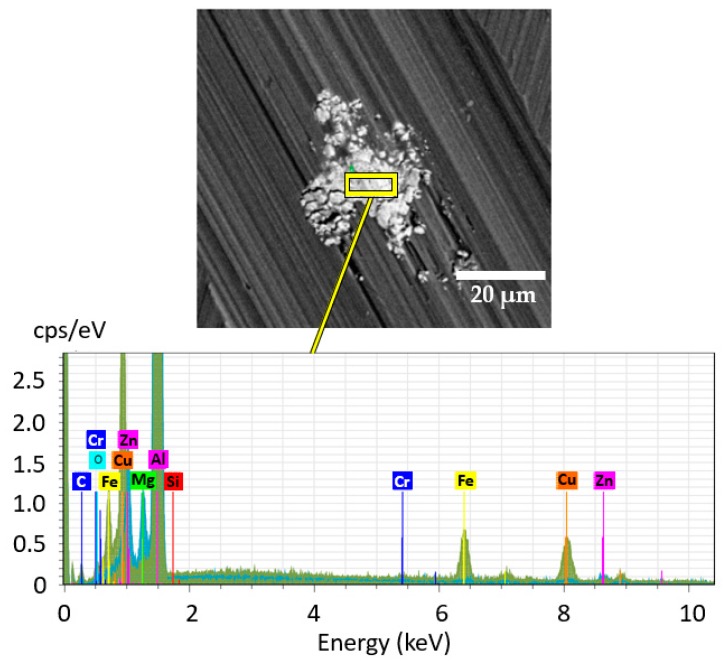
EDX of a selected intermetallic particle in alloy 7475-T7351.

Energy dispersive X-ray spectroscopy (EDX) was used for elemental analysis of the large intermetallic particles, as shown in Figure 3.

### 2.2. Tool Geometry

The end-milling whole carbide tool ⌀16×55-115 mm JHF 980 Special provided by SECO Tools company, with (Ti, Al)N coating was used for advanced high feed face milling of the specimens. To exclude any potential impact of inaccurate tool geometry, optical 3D tool geometry analysis was performed using a special software subprogram of the ALICONA-IF G5 optical microscope called “Alicona Edge Master”. The principle of this analysis was a gradual positioning of the reference plane perpendicular to the cutting edges of the tool. The measured results of the intersection of the reference plane with the cutting edges were statistically processed to obtain final results of the mean radius of the mean cutting edges and information about the mean cutting angles.

### 2.3. Surface Topography Analysis

Complex measurement of the surface topography was performed on a set of samples machined by different cutting parameters of face milling, as presented in Table 3, with the tool positioned perpendicular to the machined surface. Measurement of the surface topography for a set of samples with tool inclination of 1° was also performed. The high-resolution optical microscope ALICONA IF-G5 was used for analysis of roughness parameters (“*R*”), waviness parameters (“*W*”), Firestone–Abbott parameters, and other advanced 3D surface texture parameters (“*S*“). The measurement methodology was based on the combination of the small depth of focus of the optical system with vertical scanning. In order to perform complex detection of the surface, the high-precision optics moved vertically along the optical axis and continuously captured data from the surface. A corresponding algorithm converted the acquired sensor data into 3D information and true colour images with full depth of field [24]. Nonmeasured points in the datasets were not taken into consideration for further processing or for calculation of corresponding parameters due their low ratio (flat surfaces of samples, good fits of data with the Gaussian distribution, very low occurrence of nonmeasured points in the whole dataset). The measurement methodology was in accordance with the standard EN ISO 25178-606 [25].

### 2.4. Force Loading Analysis of High Feed Face Milling and Induced Severe Plastic Deformation

Cutting experiments for various cutting speeds and cutting feeds were carried out with a five-axis milling center MCV 1210/Sinumerik 840D. A stationary KISTLER 957B/SW dynamometer was used for measurement of the force loading during high feed face milling for the different cutting conditions, as presented in Table 4. The results have been analyzed with DynoWare software (type 2825A, Kistler, Wintherthur, Switzerland), where mean values of the maximal instantaneous force loading in the X, Y, and Z directions were used for graphical determination of the resultant force *F_1M_* and its vector decomposition to the cutting force *F_C_* and the force perpendicular *F_CN_*. The cutting force and non-deformed chip cross section *A_D_* was used for calculation of the specific cutting energy *k_c_* for a given material characterized by the constants *c_o_,* the axial depth of cut *a_p_*, the radial with of cut *a_e_*, a angular tooth engagement *ϕ* and the effect of chip thickness on specific force loading expressed with the parameter *mc*:

(1) $$k_{c}=\frac{F_{c}}{A_{D}}=\left(\frac{c_{o}}{a_{p}\times a_{e}}\right)\times \int_{\varphi 1}^{\varphi 2}sin^{1-mc}\varphi \times d\varphi$$

**Figure 4:**
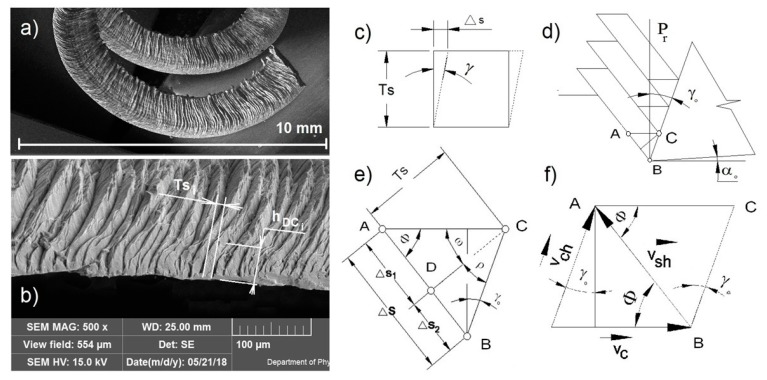
Analysis of the chip formation model when cutting: (**a**) Continuous chip; (**b**) Position of the measured lamella; (**c**) The shear deformation of the lamella; (**d**) Geometry of the cutting tool; (**e**) Model of the shear deformation in the primary zone; (**f**) Speed vector diagram.

The basic model of continuous chip formation and the individual parameters for shear deformation and rate of the deformation can be seen in Figure 4.

The plastic flow of the material is defined with the condition of a constant volume of machined material V passing through the first deformation zone and converted to the chip:

*Div* × *V* = 0 (2)

*V* = *A_D_*.× *v_c_* = *A_Dc_*× *v_ch_* (3)

where *A**_D_*** is the cross section of the undeformed material entering with cutting speed v_c_ and *A**_DC_*** is the cross section of the material converted to a chip, leaving with speed of chip v_ch_. The other variables can be understood according to Figure 4a–f.

The angle of the shear plane *φ* is defined as

(4) $$\phi =arctg\frac{sin\delta_{o}}{\Lambda -\cos\delta_{o}}$$

where the orthogonal cutting angle *δ_o_* is sum of the orthogonal flank angle and orthogonal cutting edge angle *β_o_*, i.e., *δ_o_* *=* *α_o_* *+* *β_o,_* and Λ means the chip thickness coefficient, which is expressed as

(5) $$\Lambda =\frac{h_{DC}}{h_{D}}$$

The shear deformation *γ* in the primary zone can be derived as function of shear angle φ and orthogonal rake angle *γ_o_*

(6) $$\gamma =\frac{cos_{\gamma_{o}}}{sin\phi cos(\phi -\gamma_{o})}$$

and the rate of shear deformation sequentially as

(7) $$\dot{\gamma }=\frac{cos\gamma_{o}}{cos(\phi -\gamma_{o})}\frac{v_{c}}{T_{s}}$$

The average thickness *T_s_* and thickness *h_DC_* of the material lamella can be measured and calculated statistically by electron microscopy, as seen in Figure 4, and the orthogonal rake angle *γ_o_* can be measured with the Alicona G5 microscope. The parameter *h_D_* corresponds to the feed per tooth.

### 2.5. Fatigue Testing and Frature Surface Analysis 

The objective of the fatigue testing was to examine the influence of the defined cutting conditions of the face milling on the fatigue life, as summarized in Table 5. The effect of tool inclination of 1° has also been examined.

The geometry of the fatigue specimens was chosen to achieve the best match with final operational use of the bottom wing panel. The main criteria for appropriate specimen geometry are defined as follows:

- Specimen must allow performance analysis of the effect of the high feed face milling on the fatigue life. Therefore, the flat specimen with largest possible functional area must be chosen.
- Specimen must allow simulation of the tensile cyclic loading during operation.
- Specimen must comply with ASTM E466-15 [26] and EN 6072 [27] aviation standards.

All specimens were oriented in the L-T direction of the rolled plate. Specimens were machined at the five-axis MCV 1210/Sinumerik 840D milling center (TAJMAC ZPS, share company, Zlin, Czech Republic/Siemens AG, Erlangen, Germany). Specimens were specially protected against bending or torsion during machining, and excess heating was limited by use of CIMSTAR 597 coolant (Cimcool Industrial Products B.V., Vlaardingen, the Netherlands) of 10% volume concentration, 20 bar pressure, and 20 L/min flow rate. All functional areas of the specimens have been protected against scratches, all sharp edges rounded to 0.3 mm radius, and all other stress concentrators have been removed.

Fatigue testing has been performed at special axial testing machine BISS and parameters of the testing are defined as follows:

- Fluctuating tensile cycle with stress ratio, *R* = 0.1.
- Frequency, *f* = 10 Hz.
- Stress levels: 180 MPa, 220 MPa, 250 MPa, and 300 MPa.

The source of fatigue crack nucleation was examined with the scanning electron microscope (TESCAN ORSAY HOLDING share company, Brno, Czech Republic) TESCAN MIRA 3 operating in both secondary and backscattered electron mode. The fatigue crack initiation and propagation mechanism and the integrity of the adjacent surfaces were investigated.

### 2.6. In Situ Testing

A specimen with special geometry was designed for in situ tensile mechanical testing. Profiles of the specimen were cut by EDM wire cutting (Electrical Discharge Machining) and polished. Flat functional surface areas were face milled with a special high feed end-mill (SECO tool JHF 980 Special, *f_z_* = 0.05 mm, *v_c_* = 200 m/min, *a_p_* = 0.2 mm), and all sharp edges were rounded and polished. 

**Figure 5:**
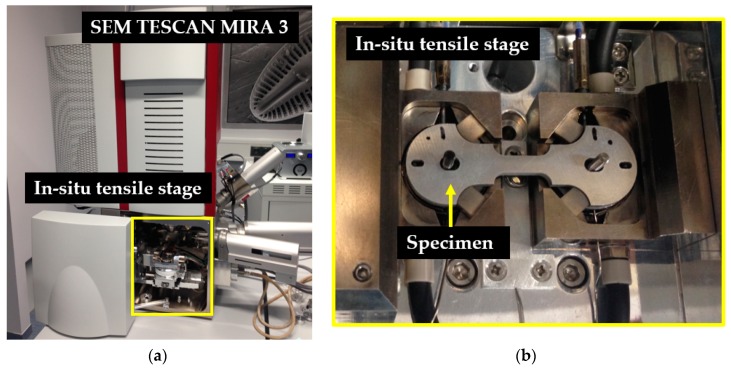
(**a**) SEM TESCAN MIRA 3 and in situ tensile stage MT1000 made by NewTec; (**b**) Specimen clamping in the in situ tensile stage.

Testing was performed on a special in situ tensile stage MT1000 made by NewTec (10 kN, a tensile stage) and all analyses were carried out with the SEM TESCAN MIRA 3, equipped with the NewTec SoftStrain software (version 1, NEWTEC, Nîmes, France), as shown in Figure 5.

#### 2.6.1. In Situ Tensile Testing

The main scope of the in situ tensile testing was to observe the crack initiation and propagation mechanism in the 7475-T7351 alloy. Two main analyses were performed: (a) Observation of the entire functional area of the specimen; and (b) detailed observation of selected intermetallic particles located at the free machined surface of the specimen. Engineering strain distribution under tensile loading in selected particle was analyzed by DIC (Digital Image Correlation) in MERCURY real-time tracking software.

#### 2.6.2. In Situ Cyclic Testing

The aim of the in situ tensile cyclic loading was to simulate cyclic loading under the operation mode and to observe crack nucleation and short crack propagation. Detailed observation of twenty selected large intermetallic particles was performed in parallel. Parameters of the fatigue loading were defined as follow:

- Stress control in the range from 35 MPa to 350 MPa (corresponding to force loading from 245 N to 2450 N).
- Stress ratio, *R* = 0.1.
- Speed of loading: 100 N/s.

## 3. Results

### 3.1. Tool Geometry

The geometry of the tool and profile roughness of the cutting edges were analyzed and the results are presented in Table 6 and Table 7. All parameters complied with manufacturer’s specifications. The standard deviations were in the range of 3–4% of the mean values.

### 3.2. Surface Topography Analysis

**Figure 6:**
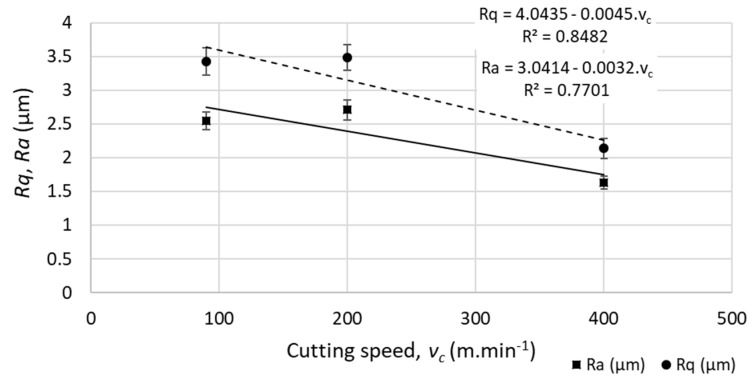
Example of profile roughness parameters (*Ra—*average roughness of the profile, *Rq*—root-mean-square roughness of the profile) for different cutting speed (at feed per tooth *f_z_* = 0.05 mm). The parameter *R^2^* reflects the level of statistical correlation.

The effect of the defined cutting conditions and tool inclination on *R*-parameters of the surface topography was evident. For the set of samples machined with tool positioning perpendicular to the machined surface, a digression of the average values of the profile roughness parameter (measured perpendicularly to the cutting speed, along the feed speed and longitudinal axis of the samples) was observed:

- The average roughness (*Ra*) and root-mean-square roughness (*Rq*) dropped by 35% for the highest cutting speed (*v_c_* = 400 m.min^−1^).
- No linear function was observed between the cutting speed and the profile roughness parameters, as indicated in Figure 6.
- Standard deviations of the repeated measurements varied between 5% and 8% of the average values for all measurements and conditions.

**Figure 7:**
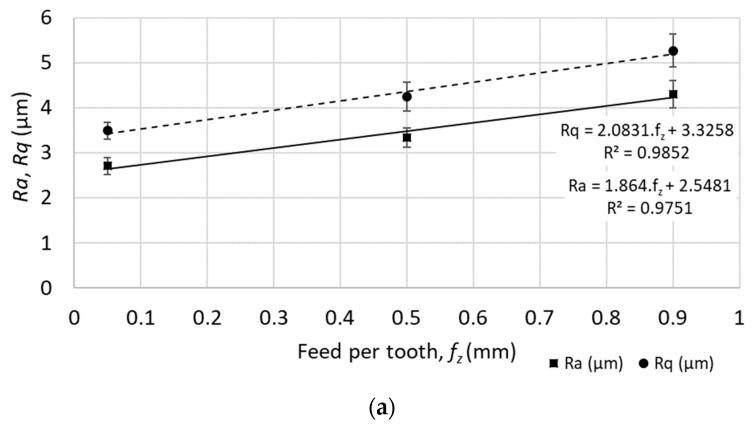

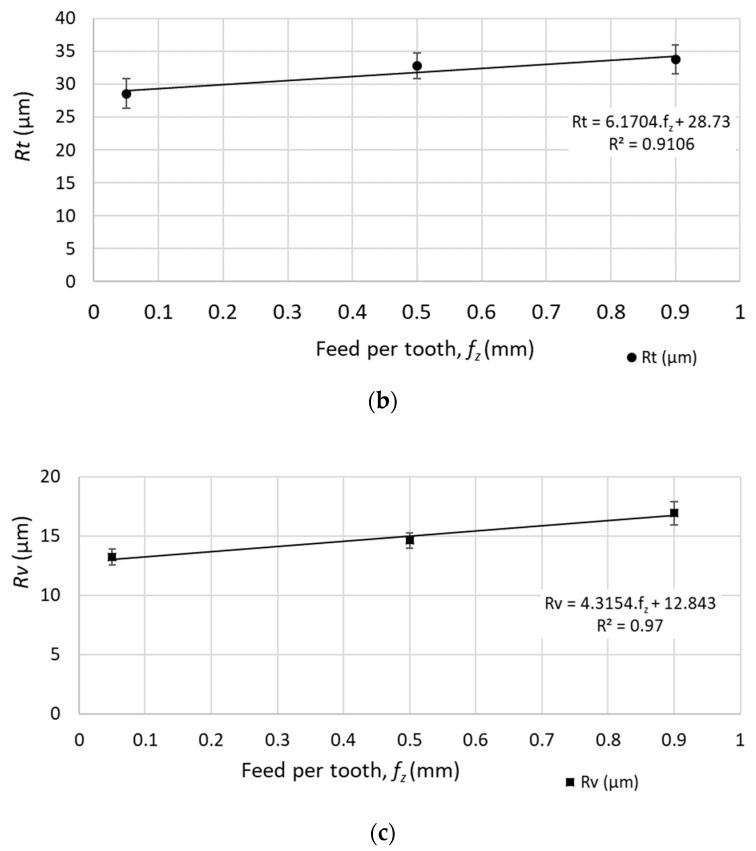
Selected profile roughness parameters for different feed speeds (at cutting speed *v_c_* = 200 m.min^−1^): (**a**) *Ra* – Average roughness, *Rq* – root-mean-square roughness; (**b**) *Rt* - maximum peak to valley height of roughness profile; (**c**) *Rv* - maximum valley depth of roughness profile.

On the other hand, the increase of the profile roughness parameters was observed with the increase of the feed speed (increase of the feed per tooth, *f_z_*, from 0.05 to 0.90 mm). Some examples of the profile roughness values increasing while increasing the feed speed are mentioned below:

- The average roughness (*Ra*) increased from 2.71 to 4.30 μm (an increase of 37%), as indicated in Figure 7.
- The root-mean-square roughness (*Rq*) increased from 3.49 to 5.27 μm (an increase of 34%), as indicated in Figure 7.
- The maximum peak to valley height of roughness profile (*Rt*) increased from 28.59 to 33.78 μm (an increase of 15%), as indicated in Figure 7.
- The maximum valley depth of roughness profile (*Rv)* increased from 13.23 to 16.92 μm (an increase of 22%), as indicated in Figure 7.

**Figure 8:**
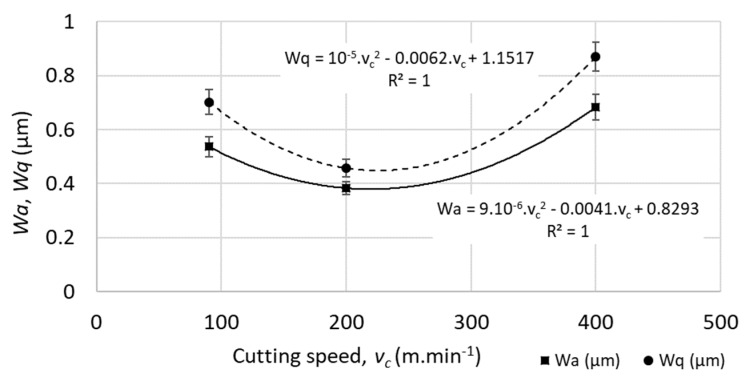
Example of profile waviness parameters (*Wa*—average waviness of the profile, *Wq*—root-mean-square waviness of the profile) for different cutting speeds (at feed per tooth *f_z_* = 0.05 mm). The parameter *R^2^* reflects the level of statistical correlation.

**Figure 9:**
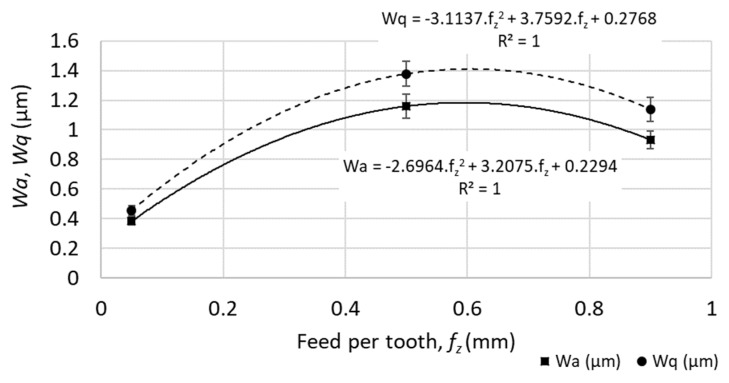
Example of profile waviness parameters (*Wa—*average waviness of the profile, *Wq*—root-mean-square waviness of the profile) for different feed speeds (at feed per tooth *v_c_* = 200 m.min^−1^). The parameter *R^2^* reflects the level of statistical correlation.

No linear function was observed between the cutting speed and the waviness parameters, or between the feed speed and the waviness parameters (*R*-squared parameter varied in the range from 0.1 to 0.7 for different waviness parameters), as shown in Figure 8 and Figure 9.

**Figure 10:**
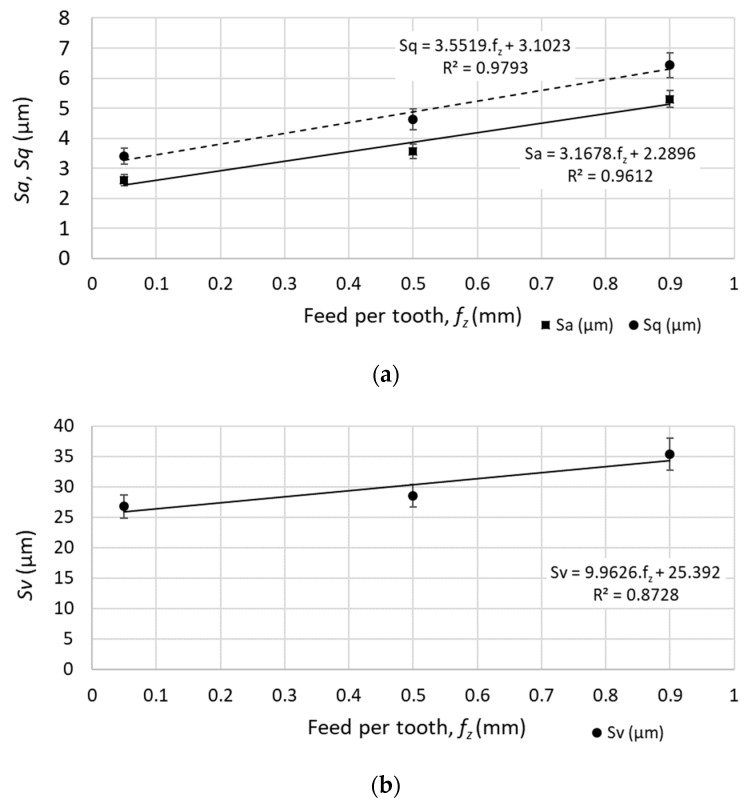
Surface parameters of the selected area for different feed speeds (at cutting speed *v_c_* = 200 m.min^−1^): (**a**) *Sa* - average height of the selected area, *Sq* - root-mean-square height of the selected area; (**b**) *Sv* - maximum valley depth of the selected area.

The surface parameters under different cutting parameters of the face milling were examined (with tool positioning perpendicular to the machined surface). The results of the measurement can be summarized as follows:

- The increase of the feed speed (increase of the feed per tooth from 0.05 to 0.90 mm) caused an increase of surface topography parameters, as indicated in Figure 10.
- The average height of the selected area (*Sa*) increased from 2.60 to 5.30 μm.
- The root-mean-square height of the selected area (*Sq*) increased from 3.40 to 6.43 μm.
- The maximum valley depth of the selected area (*Sv*) increased from 26.77 to 35.35 μm.
- No statistically significant linear function (probability 95%) was found between the cutting speed and the 3D surface topography parameters.

**Figure 11:**
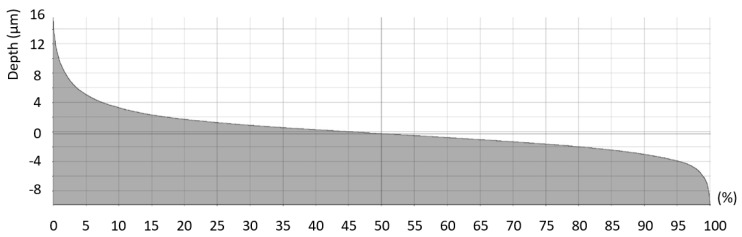
Example of Firestone–Abbott curve of the roughness profile (*f_z_* = 0.05 mm and *v_c_* = 90 m.min^−1^).

**Figure 12:**
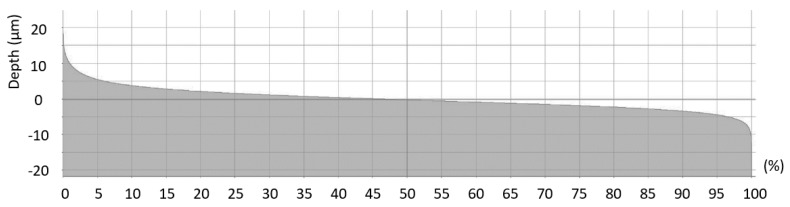
Example of Firestone–Abbott curve of the selected area (*f_z_* = 0.05 mm and *v_c_* = 90 m.min^−1^).

The machined surface can by described by the Firestone–Abbott curve, which indicates the percentage of the material of the profile elements at a defined height relative to the evaluation profile length (*R*) or surface area (*S*). This specific surface criterion is characterized by several parameters. The parameters of the core roughness depth (*Rk* and *Sk*) indicate the volume of the material above the core material which can be worn during operational use. The parameters of reduced peak height (*Rpk* and *Spk*) describe the mean height of peaks above the core material. Furthermore, the parameters of reduced peak height (*Rpk* and *Spk*) express the amount of the material that will be removed during the initial operational wearing process. The parameters of reduced valley height (*Rvk* and *Svk*) describe the mean depth of the valleys below the core material. Therefore, *Rvk* and *Svk* parameters indicate the ability of the machined surface to retain liquids. The parameter *Rmr1* indicates the fraction of the surface which consists of peaks above the core material, and the parameter *Rmr2* indicates the fraction of the surface which will carry the load [28]. An examples of the Firestone–Abbott curve are presented in Figure 11 and Figure 12.

**Figure 13:**
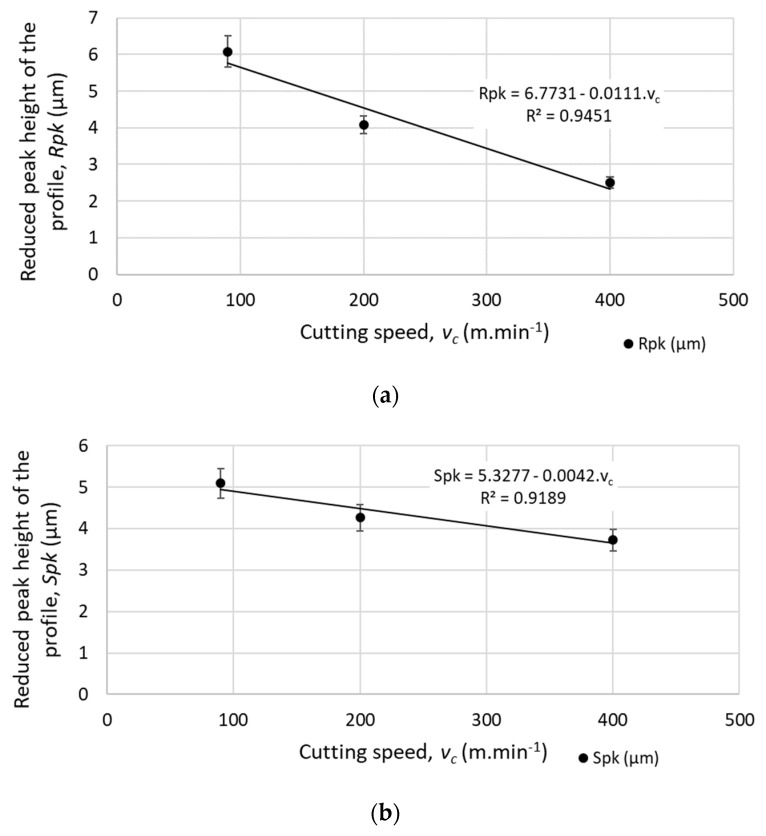
Mean height of peaks above the core material for different cutting speeds (at feed per tooth *f_z_* = 0.05 mm) (**a**) *Rpk* - mean height of peaks above the core material for a profile; (**b**) *Spk* - mean height of peaks above the core material for a selected area.

**Figure 14:**
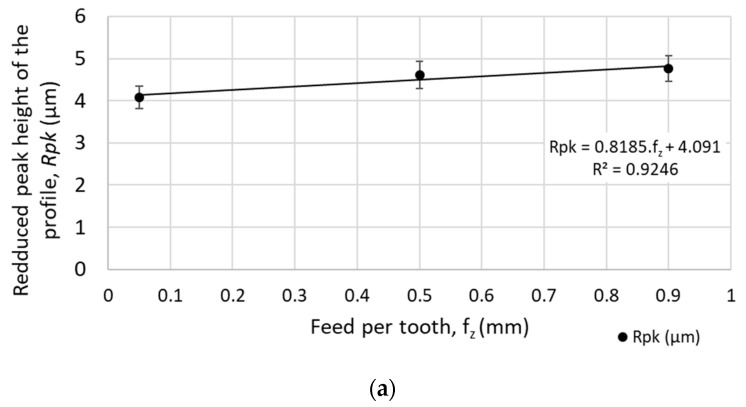

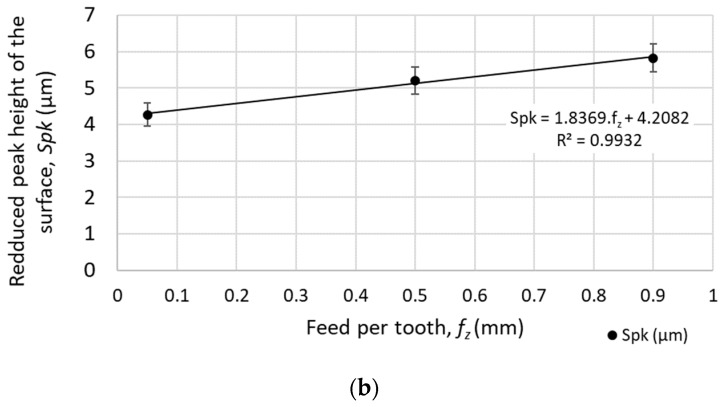
Mean height of peaks above the core material for different feed speeds (at cutting speed *v_c_* = 200 m.min^−1^): (**a**) *Rpk* - mean height of peaks above the core material for a profile; (**b**) *Spk* - mean height of peaks above the core material for a selected area.

The machined surface under the defined cutting conditions of the face milling showed the following results:

- *Rpk* and *Spk* decrease linearly with the increase of the cutting speed (while increasing the cutting speed from 90 m.min^−1^ to 400 m.min^−1^, *Rpk* dropped by approximately 58%, and *Spk* dropped by 25%), as shown in Figure 13. Therefore, less material will be removed during the initial wearing process of operational use while implementing higher cutting speeds.
- *Rk*, *Sk* and *Rpk*, *Spk* increase linearly with increasing feed speed (while increasing feed per tooth from 0.05 mm to 0.90 mm, *Rpk* increased by 16% and *Spk* increased by 26%), as shown in Figure 14. Therefore, a higher volume of the material will be removed during the initial wearing process of operational use when implementing higher feed speeds.

**Figure 15:**
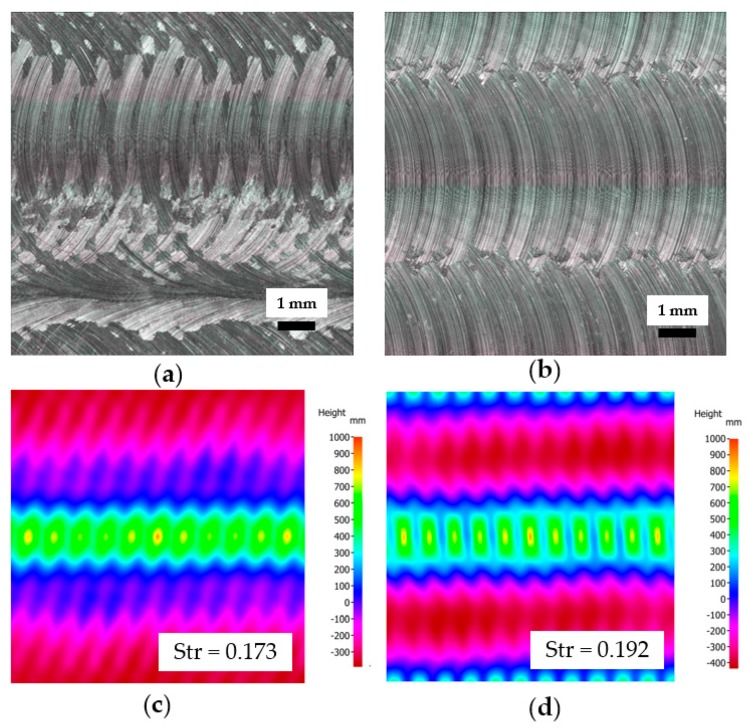
An example of the surface topography of the machined samples (*f_z_* = 0.9 mm, *v_c_* = 200 m.min^−1^, *a_p_* = 0.2 mm, *a_e_* = 6.0 mm, dry cutting): (**a**) Without a tool inclination; (**b**) Tool inclination of 1°; (**c**,**d**) Corresponding autocorrelations and texture aspect ratio values of the surface structures.

Analysis of the effect of the tool inclination on the surface parameters can be summarized as follows:

- Roughness parameters are similar for both types of strategies; however, a greater increase of roughness parameters while using higher feed speeds was observed for samples machined with tool inclined by 1°.
- Roughness parameters were affected by the feed per tooth, not by the tested cutting speeds.
- The effect of the tool inclination cannot be compared properly if no 3D surface topography parameters are used (see example of two machined samples with different tool positioning in Figure 15).
- The average height of the selected area (*Sa*) showed higher values for samples machined with the tool inclined by 1° (by 50–70%) compared to the results with perpendicular tool positioning, which were in the range of 2.00 to 5.30 μm for all tested conditions. Similar relations were found for *Sq*, *Ssk*, and *Sku*.
- The texture aspect ratios (*Str*) for the machined surface did not present significant changes (the differences for the same cutting conditions were about 15–20%).
- Roughness parameters are not sufficient for comparison of the complex topography of the surface after machining because of their inhomogeneity and sensitivity to the measured place, which affect skewness, kurtosis, etc.
- Study of the surface parameters revealed that no crucial variable of surface topography was linked to the fatigue results of the studied material.

### 3.3. Force Loading Analysis of High Feed Face Milling and Induced Severe Plastic Deformation

**Figure 16:**
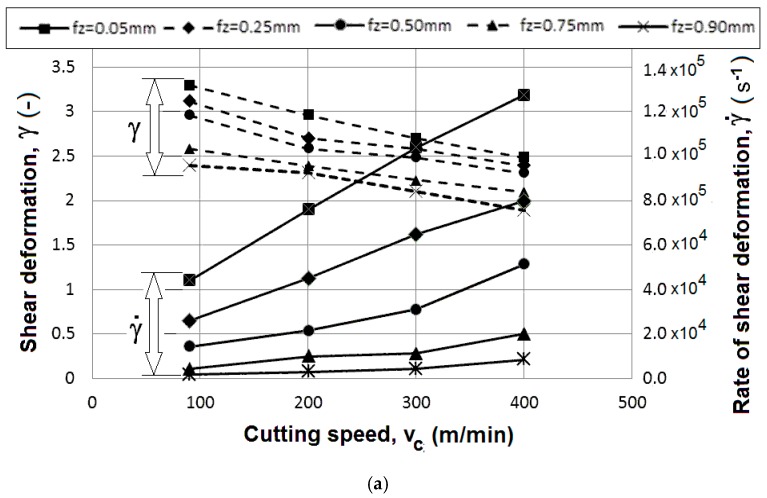

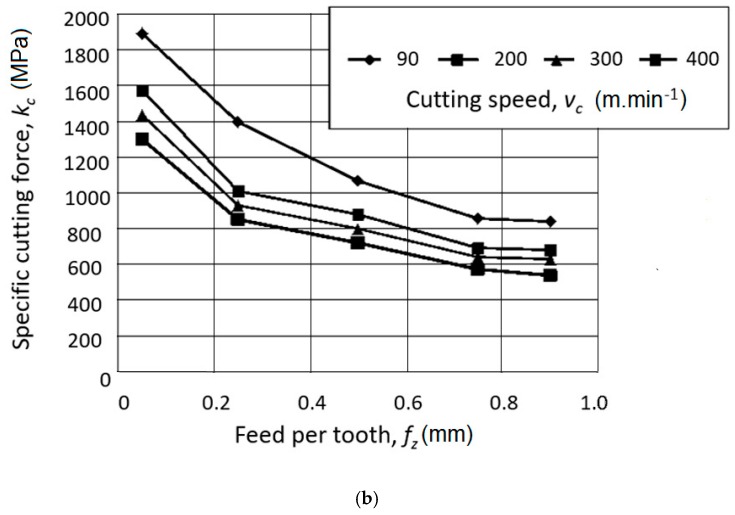
(**a**) The calculated mean values of the shear deformation and the rate of shear deformation in the primary zone; (**b**) Specific cutting forces for the given cutting conditions.

The parameters of primary shear deformation and its rates were very high, as can be seen in Figure 16. Primary shear deformation decreases with increase of the cutting speed as well as with increase of the feed speed (feed per tooth).

An explanation of this phenomenon is not easy and other works dealing with dislocations are ongoing. Meanwhile, we tentatively propose that there exists a small region over which there is a sudden proliferation of high angle boundaries in the microstructure of the material as it is deformed into the chip [29].

### 3.4. Fatigue Testing and Frature Surface Analysis

The effect of the milling parameters on the fatigue life was striking even if the surface topography after machining was not the key factor affecting the fatigue crack nucleation.

**Figure 17:**
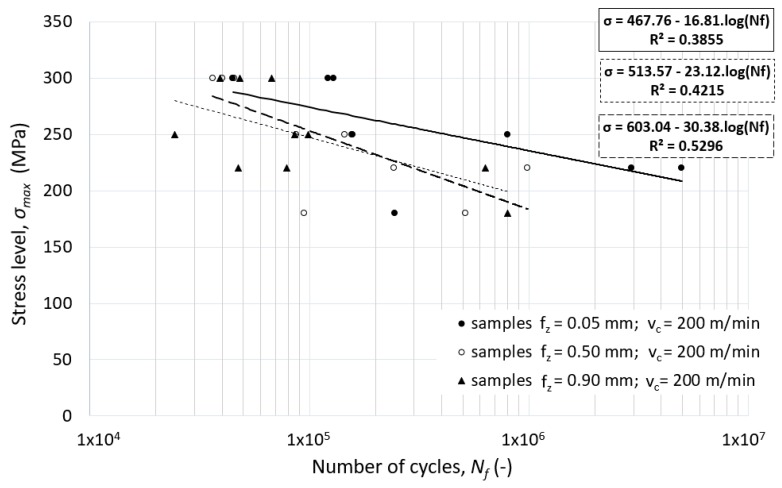
The effect of the feed speed (feed per tooth) on the fatigue life of alloy 7475-T7351—flat un-notched specimen (*v_c_* = 200 m.min^−1^).

The important decrease of the fatigue life of specimens machined with higher feed rates while keeping the same cutting speed (an increase from feed per tooth *f_z_* = 0.05 mm to *f_z_* = 0.90 mm) can be seen in Figure 17. This decrease of the fatigue life may be caused by the severe plastic deformation achieved in the smallest chip cross sections and machined at the highest cutting speeds (*v_c_* = 200 m.min^−1^).

**Figure 18:**
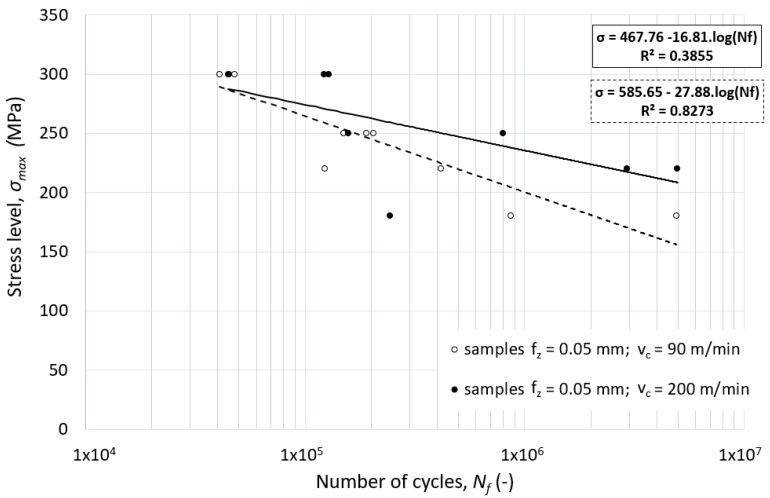
The effect of the cutting speed combined low feed cutting on the fatigue life of alloy 7475-T7351—flat un-notched specimen.

The effect of the higher cutting speed on the fatigue life of the specimen is evident in the case of combination with lower feed speed, as presented in Figure 18. Use of higher feed speed increases the fatigue life for both low and high cycle fatigue modes. However, this effect is suppressed by combination with a high feed cutting strategy.

**Figure 19:**
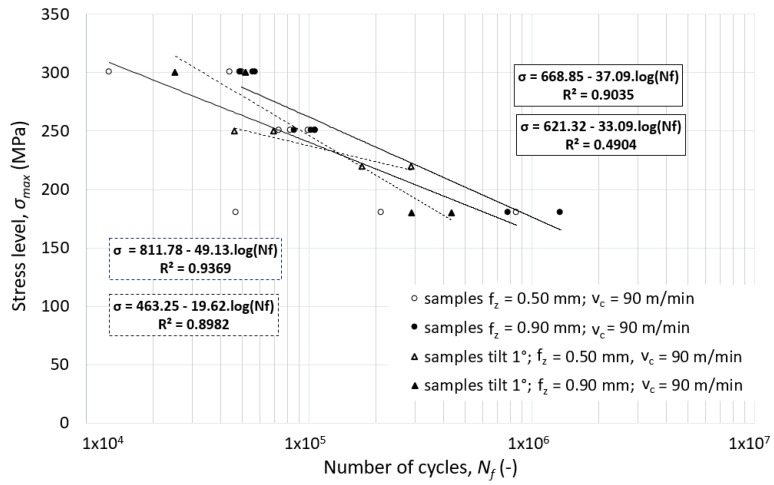
The effect of the tool inclination on the fatigue life of alloy 7475-T7351—flat un-notched specimen.

Slight inclination of the cutting tool (only 1°) resulted in a 29–64% reduction of total cycles (for specimens machined with the combination of the cutting speed *v_c_* = 90 m.min^-1^ and different feed speeds, as indicated in Figure 19).

**Figure 20:**
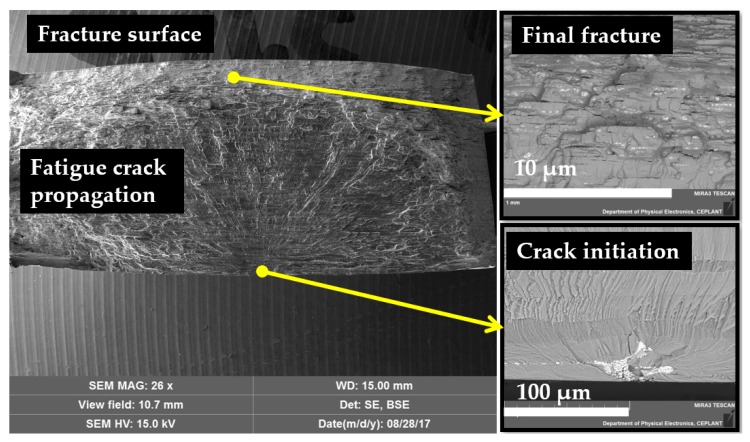
Fracture surface for a specimen machined with the following parameters: *f_z_* = 0.90 mm and *v_c_* = 90m.min^−1^, stress level 250 MPa.

Regardless of the cutting conditions, the fatigue cracks were always initiated in the large intermetallic particles which occurred in different morphologies (as large particles, elongated particles, or clusters of intermetallic particles). Fatigue cracks were mostly initiated in the intermetallic particles located in the vicinity of the machined surface regardless of the stress level. An example of the fracture surface is presented in Figure 20.

### 3.5. In Situ Testing

#### 3.5.1. In Situ Tensile Testing

**Figure 21:**
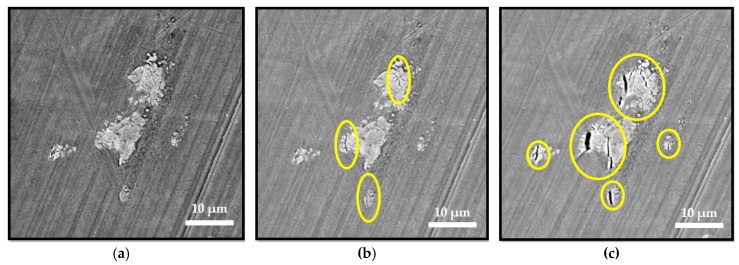
Observation of particle cracking: (**a**) Intermetallic particle before tensile loading; (**b**) Intermetallic particle at yield strength (415–419 MPa); (**c**) Intermetallic particle at tensile strength limit (484 MPa). The loading axis was horizontal.

Crack propagation from large intermetallic particles to the material matrix was observed even before reaching the tensile strength limit (484 MPa), as presented in Figure 21. Large intermetallic particles were the main source of the local stress concentration regardless of the severe plastic deformation caused by the machining process. Cracks propagated at the angle of 45° to the direction of the maximum shear stresses.

**Figure 22:**
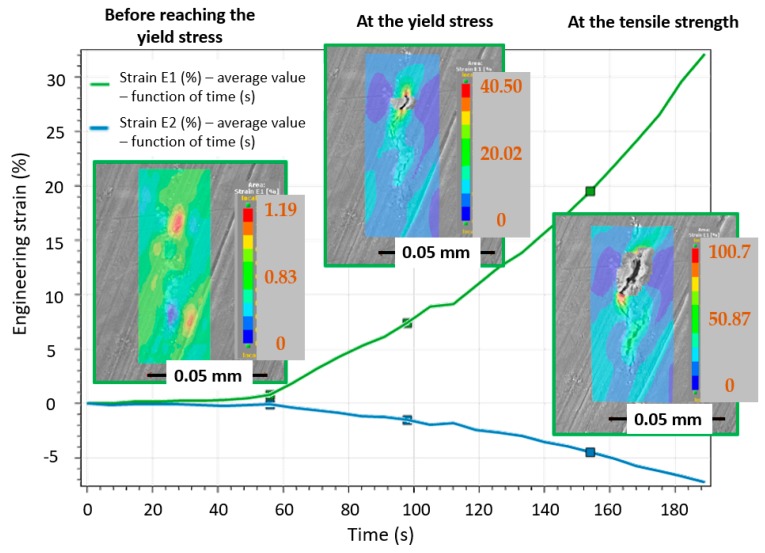
DIC analysis of the engineering strain distribution in the intermetallic particle during tensile loading: at the beginning of the tensile loading, at the yield strength, and at the tensile strength limit.

The evolution of the engineering strain distribution under tensile loading is shown in Figure 22. The average engineering strain at the area of the intermetallic particle at yield strength was in the range of 0.80% to 0.90%, and upon reaching the maximal engineering strain (1.15–1.20%), local crack initiation was observed.

#### 3.5.2. In Situ Cyclic Testing

**Figure 23:**
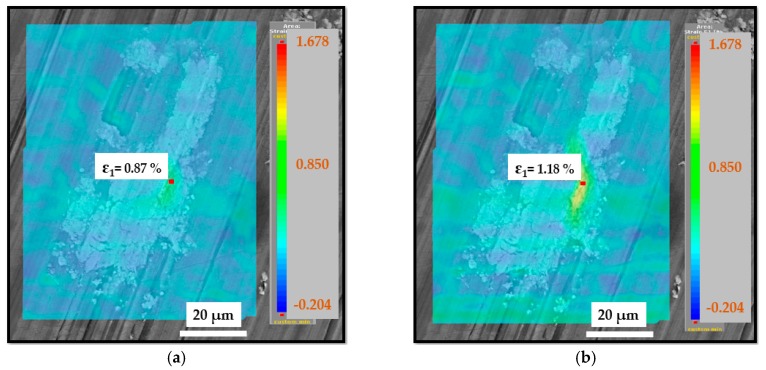
Engineering strain distribution (**a**) at 100 cycles; (**b**) at 500 cycles.

During in situ cyclic testing, initial local fatigue cracks were observed in some intermetallic particles before the fatigue life (1000 cycles) was reached, regardless of severe plastic deformation induced by the milling process. Local fatigue cracks were initiated in the core of the intermetallic particles, and with the rising number of cycles, the fatigue cracks propagated locally to the boundary of the intermetallic particles and the material matrix. Fatigue testing was interrupted at the fatigue level of 6000 cycles. Up to this number of fatigue cycles, the short fatigue cracks remained inside the intermetallic particles and did not propagate further. DIC analysis confirmed that once the local engineering strain reached values of 1.15–1.20% (at 1000 cycles, in this case), the local fatigue cracks were initiated, as demonstrated in Figure 23.

## 4. Discussion

The observations from the experimental machining, surface analyses, and fatigue testing confirm similar results as those of Ojolo et al. [17] and Novovic et al. [18].

Surface topography analysis confirmed that the roughness parameters increase with the increase of the feed speed (feed per tooth). The increase of the cutting speed caused a decrease of the surface roughness parameters. This result partially confirms the observation of Ojolo et al. [17].

The increase of the feed speed increases surface topography parameters such as average height of the selected area (*Sa*), root-mean-square height of the selected area (*Sq*), or maximum valley depth of the selected area (*Sv*). The roughness parameters were found to be similar for both strategies (perpendicular and inclined by 1°); however, a greater increase of roughness parameters was observed while using higher feed speeds for samples machined with the tool inclined by 1°. The average height of the selected area (*Sa*) showed higher values for samples machined with the tool inclined by 1°. This parameter was not adequate, considering that maximum valley depth of the selected area (*Sv*) was higher for samples machined by a tool positioned perpendicularly to the machined surface.

The trends of specific cutting force and shear deformation confirm a reduction of plastic deformation with increasing cutting speed, but more intensive deformation with reduction of feed per tooth. In other words, the intensity of plastic deformation is higher for shallow cuts and higher cutting speeds.

The highest fatigue resistance was observed at samples machined with the highest cutting speed (*v_c_* =200 m.min^−1^) and lowest feed per tooth (*f_z_* = 0.05 mm). A decrease of the fatigue life of specimens machined with higher feed rates while keeping the same cutting speed was observed (increase from feed per tooth *f_z_* = 0.05 mm to *f_z_* = 0.90 mm). This decrease of the fatigue life may be caused by the severe plastic deformation achieved in the smallest chip cross sections and machined at the highest cutting speeds (*v_c_* = 200 m.min^−1^).

Slight inclination of the cutting tool (1°) resulted in reduction of the total cycles for specimens machined with cutting speed *v_c_* = 90 m.min^−1^ and different feed speeds. This reduction may be caused by plastic deformation caused by teeth not engaged in the cut, as in the case of face milling perpendicular to the machined surface. This plastic deformation may impose beneficial compressive residual stresses into the machined surface and thus increase the fatigue life.

Therefore, the cutting conditions can affect the material removal rate, but a more serious impact can be seen in terms of the surface quality and the resistance to mechanical loading. The effect of inclusions is very serious, and materials used for dynamic loading should be carefully analyzed not only in view the surface integrity, but also considering the occurrence of the phases, which confirms the results of Piska et al. [20]. The effect of material hardening and thermal softening when cutting should be studied further in terms of the density and arrangement of dislocations, stacking fault energy, and other atomic hardening or softening mechanisms.

## 5. Conclusions

The application of very advanced laboratory facilities yielded the following results:

- The 7475-T7351 aluminum material was suitable for dynamic mechanical loading with good machinability when milling with the special monolithic cutter SECO JHF 980 Special.
- The quality of surface parameters and fatigue resistance improved when higher cutting speeds (200 m/min) and low feeds per tooth (0.05 mm) were used, and extreme shear deformation (*γ* = 2.5) and deformation rates (1.2 × 10^5^ s^−1^) were achieved.
- The measured values of *Sa* correlated with the *Ra* parameters in the trends according to the cutting conditions and proved to be featureless multiplications of the *Ra* values for all tested conditions. Nevertheless, no significant variable of the surface topography linked to the fatigue results was found.
- The fatigue resistance of the samples machined with the standard perpendicular position of the tool to the machined surface (i.e. without any spindle inclination) was greater than the results for samples machined with the inclined tool. Therefore, surfaces with more complex topography seems to be beneficial; however, new studies with a material with minimal intermetallic inclusions are needed.
- The crucial and decisive factor for crack nucleation can be seen in the coarse intermetallic inclusions (Al_7_Cu_2_Fe; Al-Cr-Fe-Cu-Si) in sizes from 2 µm up to 20 µm, suppressing the effect of the surface parameters after machining.
- Further study of the dislocation mechanism responsible for deformation hardening and softening are required. Research relating surface layer depths and grain size are ongoing.

## Figures and Tables

**Table 1:** Chemical composition of the 7475-T7351 alloy (in wt.%) [4].

Si	Fe	Cu	Mn	Mg	Cr	Zn	Ti	Other, Each	Al
0.10 max	0.12 max	1.20–1.90	0.06 max	1.90–2.60	0.18–0.25	5.20–6.20	0.06 max	<0.05	Balance

**Table 2:** Mechanical properties of the 7475-T7351 alloy [4].

Thickness of the Blank Sheet (mm)	25–38	50–63	75–89
Tensile strength (MPa)	490	476	448
Yield strength (MPa]	414	393	365
Elongation (%]	9	8	8

**Table 3:** Combinations of cutting parameters of high feed face milling.

*a_p_* = 1.50 mm; *a_e_* = 8.00 mm		Feed per tooth, *f_z_* (mm)		
**Cutting speed, *v_c_* (m.min^−1^)**	-	**0.05**	**0.50**	**0.90**
	**90**	Combination 1	Combination 2	Combination 3
	**200**	Combination 4	Combination 5	Combination 6
-	**400**	Combination 7	N/A	N/A

*a_p_—* axial depth of cut, *a_e_*— radial depth of cut, *f_z_* — feed per tooth

**Table 4:** Set of specimens used for force loading analysis.

*a_p_* = 1.50 mm; *a_e_* = 8.00 mm		Feed per tooth, (*f_z_* mm)				
**Cutting Speed, *v_c_* (m.min^−1)^**	-	**0.05**	**0.25**	**0.50**	**0.75**	**0.90**
	**90**	Combination 1	Combination 2	Combination 3	Combination 4	Combination 5
	**200**	Combination 6	Combination 7	Combination 8	Combination 9	Combination 10
	**300**	Combination 11	Combination 12	Combination 13	Combination 14	Combination 15
	**400**	Combination 16	Combination 17	Combination 18	Combination 19	Combination 20

**Table 5:** Combinations of cutting parameters of high feed face milling.

*a_p_* = 1.50 mm; *a_e_* = 8.00 mm		Feed per tooth, *f_z_* (mm)		
**Cutting Speed, *v_c_ (*m.min^−1^)**	-	**0.05**	**0.50**	**0.90**
	**90**	Combination 1	Combination 2	Combination 3
	**200**	Combination 4	Combination 5	Combination 6

**Table 6:** SECO end-milling tool ⌀16×55-115 mm JHF 980 Special geometry.

Cutting Edge	Cutting Edge Radius *r_n_* (μm)	Orthogonal Clearance Angle *α_o_* (°)	Orthogonal Edge Angle *β_o_* (°)	Orthogonal Rake Angle *γ_o_* (°)
1.	7.58	9.00	65.57	15.43
2.	7.46	9.31	65.42	15.28
3.	7.48	9.28	65.18	15.54
4.	7.45	9.09	65.19	15.72
5.	7.46	9.07	65.29	15.61

**Table 7:** SECO end-milling tool ⌀16×55-115 mm JHF 980 Special surface profile roughness parameters.

Cutting Edge	*Ra* (μm)	*Rq* (μm)	*Rz* (μm)	*Rp* (μm)	*Rv* (μm)
1.	0.29	0.37	0.67	0.37	0.63
2.	0.27	0.34	0.86	0.34	0.69
3.	0.24	0.31	0.75	0.31	0.52
4.	0.21	0.27	0.44	0.27	0.52
5.	0.25	0.30	0.59	0.32	0.54

*Ra—*average roughness of the profile, *Rq—*root-mean-square roughness of the profile, *Rz*—mean peak to valley height of the roughness profile, *Rp*—maximum peak height of the roughness profile, *Rv*—maximum valley depth of the roughness profile.
